# The efficiency of periodontal endoscopy in non-surgical periodontal therapy: a systematic review and meta-analysis

**DOI:** 10.3389/fdmed.2025.1681020

**Published:** 2025-11-24

**Authors:** Shahad B. Alsharif, Nour Hobani

**Affiliations:** Department of Periodontology, Faculty of Dentistry, King Abdulaziz University, Jeddah, Saudi Arabia

**Keywords:** periodontal disease, periodontitis, scaling and root planing, endoscopy, systematic review, meta-analysis

## Abstract

**Objective:**

Periodontal endoscopy offers a minimally invasive method to enhance subgingival visualization, potentially improving the outcomes of periodontal therapy. This systematic review and meta-analysis aim to evaluate the current evidence in the adjunctive use of periodontal endoscopy.

**Methods:**

The protocol was registered with the International Prospective Register of Systematic Reviews “PROSPERO”, managed by the Center for Reviews and Dissemination, the National Institute for Health Research, University of York, UK, under registration identification number (CRD420251051901). PubMed/MEDLINE, Google Scholar, The Cochrane Library, and ProQuest databases were searched up to June 2025 for randomized clinical trials (RCTs) published in English investigating non-surgical periodontal therapy with and without the adjunctive use of periodontal endoscopy. The authors independently with consensus extracted clinical outcomes. The RoB2, the revised Cochrane risk of bias tool for RCTs, was utilized to evaluate risk of bias. Meta-analysis was performed for quantitative assessment.

**Results:**

Nine RCTs were identified. The narrative results of the reported clinical outcomes were diverse. Meta-analysis revealed that periodontal endoscopy was associated with significantly less residual calculus (mean difference: −1.87%, *p* = 0.0010, I2 = 0%), significantly longer treatment time by 6.01 min (*P* < 0.00001, I2 = 0%), and greater probing depth reduction (mean difference: −0.47, *p* = 0.004, I2 = 94%).

**Conclusion:**

The adjunctive use of periodontal endoscopy outperformed the conventional scaling and root planing alone in calculus removal but appears to be more time-consuming. Yet, more homogeneous RCTs are necessary to attain clear evidence on additional clinical outcomes.

**Systematic Review Registration:**

https://www.crd.york.ac.uk/PROSPERO/view/CRD420251051901, Identifier CRD420251051901.

## 1 Introduction

Periodontal disease is a chronic infectious disease that leads to the damage of the structures of the periodontium; these consist of the periodontal ligament, alveolar bone, and gingiva, which can eventually result in tooth loss (1). These destructive changes are caused by dysbiosis due to the presence of bacterial plaque. Bacterial plaque is the primary etiologic factor, while mineralization into calculus is considered a secondary etiologic factor, as it harbors the bacteria and provides an environment to colonize and metabolize, leading to disease progression (2).

The primary purpose of periodontal therapy is the elimination of the bacterial plaque and its byproducts as well as the calculus deposits, hence achieving a biologically acceptable root surface and arresting the progression of the inflammatory process, thus preserving the periodontium and dentition (3). Scaling and root planing (SRP) is the main part of non-surgical periodontal therapy; however, calculus is firmly attached to root surfaces through different means of attachments and mechanical interlocking, which makes it challenging to achieve complete removal, particularly in areas where complex anatomical factors are present (4, 5).

Conventional non-surgical periodontal therapy involves SRP utilizing manual curettes and different types of powered scalers with traditional means of calculus removal evaluation with tactile exploration. One main limitation of this conventional approach is the failure to completely remove the calculus as well as to detect the left-behind deposits. Residual calculus was shown to be left-behind by both experienced and inexperienced clinicians, particularly in periodontal pockets ≥4 mm, at the cemento-enamel junction, in furcation invasions, and in association with anatomical grooves (6). Studies demonstrated that only 32% of calculus-free surfaces were achieved in periodontal pockets of >6 mm after conventional SRP (6), multi-rooted teeth were more difficult to debride completely, with 60% of molar teeth exhibiting residual calculus (7). As a result, different methods to improve calculus visualization and removal have been developed.

General surgeons employed endoscopy many years ago to visualize through openings and channels in minimally invasive surgeries. Gradually, the use of this endoscopic technology was expanded from medical uses, reaching different fields in dental and maxillofacial; these involve arthroscopy for temporomandibular joint (8), maxillary sinus endoscopy (9), root canal endoscopy (10), and periodontal endoscopy (11).

Periodontal endoscopy enhances the visibility of the subgingival environment non-surgically without the need for periodontal flap reflection, as this procedure allows the visualization of the root surfaces with high magnification (12). The periodontal endoscopy system consists of an endoscope that allows root surface illumination and magnification by a fiber optic bundle. This fiber bundle is attached to an explorer, and upon inserting the explorer into the gingival sulcus, the image is transmitted to the screen, which can be seen by the clinician in real time. This explorer is used simultaneously with an ultrasonic scaler or curette while performing SRP, resulting in better visibility of the subgingival pockets, particularly calculus, thus better calculus elimination results (11, 13).

Research has demonstrated that SRP with the adjunctive use of a periodontal endoscope presented benefits over the conventional method in different clinical parameters, less bleeding on probing and gingival inflammation (14), and less residual calculus after SRP with a periodontal endoscope as opposed to conventional SRP (15). However, other investigations stated contradicting findings, as they reported no significant difference between periodontal endoscopy-aided SRP and the conventional method (16, 17).

Therefore, due to these conflicting results, a need for a systematic review and meta-analysis is necessary to provide better understanding and analysis of the existing evidence regarding this matter. This systematic review and meta-analysis aims to investigate whether the adjunctive use of periodontal endoscopy in SRP has superior clinical outcomes compared to the conventional method of SRP.

## 2 Methods

### 2.1 Protocol registration

The research protocol was registered with the International Prospective Register of Systematic Reviews “PROSPERO”, managed by the Center for Reviews and Dissemination, the National Institute for Health Research, University of York, UK, under registration identification number (CRD420251051901). The investigation method was performed based on the Cochrane Handbook of Systematic Reviews of Interventions (18) and complied with the Preferred Reporting Items for Systematic Reviews and Meta-Analysis “PRISMA” (19).

### 2.2 PICO question

Following PRISMA guidelines (20), Patient, Problem, or Population; Intervention; Comparison, Control, or Comparator; Outcome (PICO) question was: “Is the adjunctive use of periodontal endoscopy in non-surgical periodontal therapy superiorly effective in comparison to conventional SRP alone?”

- Population: Systematically healthy adult patients (≥18 years old) with periodontal disease.
- Intervention: non-surgical periodontal therapy, SRP, with the adjunctive use of periodontal endoscopy.
- Comparison: conventional non-surgical periodontal therapy (SRP alone).
- Outcome(s): percentage of residual calculus and average treatment time, in addition to different clinical parameters involving probing depth (PD), gingival index (GI), bleeding on probing (BOP), and clinical attachment loss (CAL).

### 2.3 Inclusion and exclusion criteria

Inclusion criteria were randomized clinical trials (RCTs) of systematically healthy adult patients diagnosed with periodontal diseases underwent non-surgical periodontal therapy, SRP, with and without the adjunctive use of periodontal endoscopy, published in the English language. No restrictions applied regarding study timeline and follow-up or publication year.

Exclusion criteria were study designs other than RCTs. RCTs published in a non-English language. Additionally, RCTs involved the usage of periodontal endoscopy combined with a surgical approach or antimicrobial intervention.

### 2.4 Search strategy

The literature search involved the following electronic databases: PubMed/MEDLINE, Google Scholar, and The Cochrane Library with no publication year restriction up to June 2025. An extended “gray literature” search was executed utilizing ProQuest. The electronic search was enhanced by a manual review of the reference list of the selected articles.

The Medical Subject Heading (MeSH) terms and keywords were chosen according to the PICO framework of this investigation, using “AND” and “OR” adapted for each database. Details are in Table 1.

**Table 1:** Detailed search strategy for each database.

Database	Search strategy
PubMed/MEDLINE	([periodontal disease OR Gingival Disease OR periodontitis OR gingivitis OR periodontal attachment loss OR periodontal inflammation OR Sub gingival calculus OR dental calculus OR dental plaque (MeSH Terms)] AND [non-surgical periodontal therapy OR periodontal therapy OR scaling and root planning OR SRP OR subgingival scaling OR subgingival debridement OR dental scaling OR root planing (MeSH Terms)] AND [periodontal endoscopy OR perioscopy OR perioscope OR periodontal endoscope OR endoscopy OR vedioscope (MeSH Terms)] AND [patients OR adults OR humans (MeSH Terms)])
The Cochrane Library	periodontal disease OR Gingival Disease OR periodontitis OR gingivitis OR periodontal attachment loss OR periodontal inflammation OR Sub gingival calculus OR dental calculus OR dental plaque AND non-surgical periodontal therapy OR periodontal therapy OR scaling and root planning OR SRP OR subgingival scaling OR subgingival debridement OR dental scaling OR root planing AND periodontal endoscopy OR perioscopy OR perioscope OR periodontal endoscope OR endoscopy OR vedioscope AND patients OR adults OR humans
Google Scholar	(“periodontal disease” OR periodontitis OR “subgingival calculus” OR “dental calculus”) AND (“non-surgical periodontal therapy” OR “scaling and root planing” OR “subgingival scaling” OR “subgingival debridement”) AND (“periodontal endoscopy” OR perioscopy OR perioscope OR “periodontal endoscope”) AND (patients OR adults OR humans)
ProQuest	(“periodontal disease” OR periodontitis OR “subgingival calculus” OR “dental calculus”) AND (“non-surgical periodontal therapy” OR “scaling and root planing” OR “subgingival scaling” OR “subgingival debridement”) AND (“periodontal endoscopy” OR perioscopy OR perioscope OR “periodontal endoscope”) AND (patients OR humans)

### 2.5 Studies selection

Two reviewers, the authors, SBA and NH, independently assessed the obtained studies for eligibility selection following the proposed inclusion and exclusion criteria. After importing the obtained studies from all databases into Microsoft Excel (Microsoft Ltd, Washington), the duplicates were eliminated. Initially, the titles and abstracts were read to assess each study's qualification for inclusion. The study was eliminated if both reviewers concurred on the exclusion. However, when there was a discrepancy, the full manuscript was evaluated. Next, full manuscripts were examined to confirm their eligibility for inclusion and to eliminate the studies that failed to fulfill the criteria. At this stage, in case of a discrepancy between the two reviewers, it was addressed by appointing a third reviewer to reach consensus. The inter-rater agreement between the two reviewers on study selection was evaluated using Cohen's kappa statistic, revealing an almost perfect agreement (k > 0.90) (21).

### 2.6 Data extraction

The same two reviewers carried out data extraction independently. Then, the acquired information was compared for accuracy. In case of discrepancies, a discussion was carried out until a consensus was reached. Full manuscripts were reviewed to collect information on authorship and publication, participants' demographics, clinical examination specifics, intervention, post-intervention outcomes, and any follow-up results, considering all outcome variables assessed in this investigation. “Not applicable” was declared in case of any missing information with no assumptions.

### 2.7 Risk of bias in individual studies

The authors individually evaluated the risk of bias in the selected studies following the RoB2: the revised Cochrane risk of bias tool for randomized trials (22). In case of discrepancy, a discussion was made until agreement. The tool includes five domains: randomization process, deviations from intended interventions, missing outcome data, measurement of the outcome, and selection of the reported result. After assigning low, some concern, or high risk of bias to each domain, a total risk is owed for each study.

### 2.8 Meta- analysis

Meta-analysis was carried out utilizing RevMan software (The Cochrane Collaborative, version 9.6.0, Cochrane IMS). A random effect of changes in the reported outcomes (residual calculus, probing depth, and treatment time) was used to estimate the effect size and mean difference with a 95% confidence interval. Heterogeneity was evaluated utilizing a forest plot, Cochran's *Q*-test, and the I^2^ statistic.

## 3 Results

### 3.1 Search results

**Figure 1:**
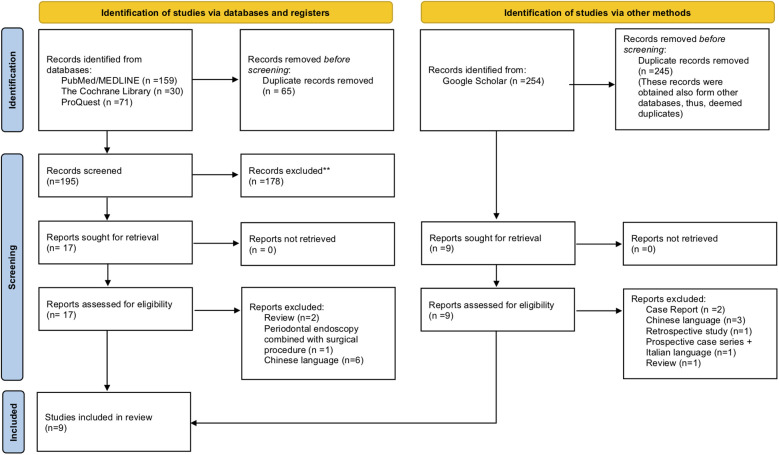
PRISMA flowchart for the databases search process and reason for exclusion.

Based on the search strategy illustrated in Table 1, the electronic search acquired a total of 514 articles from various databases: PubMed/MEDLINE (*n* = 159), the Cochrane Library (*n* = 30), Google Scholar (*n* = 254), and ProQuest (*n* = 71). After discarding the duplicates and inspecting the titles and abstracts following the specified inclusion criteria, 26 articles were selected for full article review. Additional evaluation of the full texts resulted in the exclusion of 17 articles, with a total of 9 RCTs included in this study. Figure 1 and Table 2 illustrate the detailed search result with the explanation behind exclusion.

**Table 2:** The excluded studies and the reason for the exclusion.

The excluded study	Reason for exclusion
Grzywacka K, Górski B. Subgingival mechanotherapy using a perioscope - case report. Prosthodontics. (2024) 74:137–143.	Case report
Harrel SK, Wilson TG Jr, Rivera-Hidalgo F. A videoscope for use in minimally invasive periodontal surgery. J Clin Periodontol. (2013) 40 (9):868–874.	Review
Hou Y. Comparison of the effects of periodontal scaling under the periodontal endoscope and traditional periodontal scaling on periodontitis. Chin J Pract Med. (2016) 43:68–70.	Chinese language
Kwan JY. Enhanced periodontal debridement with the use of micro ultrasonic, periodontal endoscopy. J Calif Dent Assoc. (2005) 33 (3):241–248.	Review
Kwan J, Workman P. Micro ultrasonic endoscopic periodontal debridement: Retrospective analysis of treatment with at least 1 year follow-up. J Periodontol. (2009) 80:1901–1904	Retrospective study
Li LJ, Yan X, Yu Q, Yan FH, Tan BC. Multidisciplinary non-surgical treatment of advanced periodontitis: A case report. World J Clin Cases. (2022) 10 (7):2229–2246.	Case report
Liao YT, Liu Y, Jiang Y, Ouyang XY, He L, An N. [A clinical evaluation of periodontal treatment effect using periodontal endoscope for patients with periodontitis: a split-mouth controlled study]. Zhonghua Kou Qiang Yi Xue Za Zhi. (2016) 51:722–727.	Chinese language
Lu WQ, Zhang WL, Xiang WJ, Liu BY, Cao WY, Yang YM. [Clinical effectiveness evaluation of gingival recession by using perioscope as an adjunct to non-surgical periodontal therapy]. Shanghai Kou Qiang Yi Xue. (2022) 31 (4):435–438.	Chinese language + evaluation of gingival recession
Montevecchi M, Checchi V, Simona S, Breschi L. Endoscopic-assisted non surgical periodontal therapy: radiographic evaluation of infrabony defect response. A pilot study. In: Programma 2012 XIC Congresso Nazionale del Collegio dei Docenti di Odontoiatria “L'high tech come supporto alla ricerca, alla didattica ed alla clinica in odontostomatologia”. (2012)	Prospective case series + Italian language
Pei XY, Yang W, Ouyang XY, Sun F. [Comparison of clinical effects between periodontal endoscopy aiding subgingival debridement and flap surgery]. Beijing Da Xue Xue Bao Yi Xue Ban. (2023) 55 (4):716–720.	Chinese language
Rathod AD, Jaiswal PG, Masurkar DA. Enhanced Periodontal Debridement with Periodontal Endoscopy (Perioscopy) for Diagnosis and Treatment in Periodontal Therapy. J Clin Diag Res. (2022) 16:13.	Review
Shi JH, Xia JJ, Lei L, Jiang S, Gong HC, Zhang Y, et al. [Efficacy of periodontal endoscope-assisted non-surgical treatment for severe and generalized periodontitis]. Hua Xi Kou Qiang Yi Xue Za Zhi. (2020) 38:393–397.	Chinese language
Su Q, Wang N, Zhang M, Wu J. Clinical efficacy of periodontal endoscopy-assisted subgingival scaling and root planning and its effect on psychology and quality of life in patients with periodontitis. J Prev Treat for Stomato Dis. (2024) 32:50–56.	Chinese language
Xu YJ, Zhao L, Wu YF, Duan DY. Clinical study of periodontal endoscope-assisted subgingival scaling in the treatment of residual pocket. Hua Xi Kou Qiang Yi Xue Za Zhi. (2021) 39 (4):441–446.	Chinese language
Yang Z, Wang J, Lei L, Li H. Two-year follow-up of the outcomes of endoscope-assisted minimally invasive nonsurgical periodontal therapy for deep intrabony defects. J Prev Treat for Stomato Dis. (2024) 32:350–358.	Chinese language
Zhang YH, Li HX, Yan FH, Tan BC. [Clinical effects of scaling and root planing with an adjunctive periodontal endoscope for residual pockets: a randomized controlled clinical study]. Hua Xi Kou Qiang Yi Xue Za Zhi. (2020) 38 (5):532–536.	Chinese language
Dunegan KA, Deas DE, Powell CA, Ruparel NB, Kotsakis GA, Mealey BL. Subgingival scaling and root planing during minimally invasive periodontal surgery: A randomized controlled split-mouth trial. J Periodontol. (2024) 95 (1):9–16.	Periodontal endoscopy combined with surgical procedure

### 3.2 Characteristics of included studies

All the studies were RCTs, as specified in the predefined inclusion criteria (14–17, 23–27). Further characteristics are presented in Table 3. These RCTs had comparable methodology involving two trial groups: SRP alone or periodontal endoscopy-aided SRP. Most of them utilized intraoral evaluation of different clinical parameters to assess the outcome except for two studies where the trial was performed intraorally on hopeless teeth to be extracted, and then the extracted teeth were examined under a microscope for outcomes assessment (15, 17).

**Table 3:** Characteristics of the included studies.

Author, Year	Country of the study	Study design	Inclusion criteria	Patients	Gender
Wright et al. (2023) (23)	USA	RCT (Split mouth)	Age ≥18 years, good general health, diagnosis of generalized stage II or stage III periodontitis as per the 2017 World Workshop classification, required whole mouth SRP, maximum of 6 mm PD, at least one molar in each arch, excluding third molars	Initial: 25 1 month: 23 3 months: 23 6 months: 19 12 months: 11	Initial: 9M/16F 3 months: 8M/15F 6 months: 6M/12F 12 months: 4M/5F
Michaud et al. (2007) (17)	USA	RCT (Tooth pairs)	Patients had at least two multirooted first or second molars with non-fused roots having a hopeless periodontal or restorative prognosis and at least one site with a PD ≥5 mm	24 patients	11M/13F
Blue et al. (2013) (14)	USA	RCT (Split mouth, Pilot)	Healthy adult volunteers, ≥18 years old with chronic moderate periodontitis, required to have at least 4 sites with PD of 5–8 mm in each of 2 quadrants	Initial: 30 patients Completed the study: 26 patients	19M/7F
Geisinger et al. (2007) (15)	USA	RCT (Tooth pairs)	Patients had at least two single-rooted (incisor, canine, or premolar) teeth with a hopeless periodontal or restorative prognosis and at least one site with PD ≥5 mm	15 patients	6M/9F
Naicker et al. (2022) (24)	Australia	RCT (Two groups)	Subjects diagnosed with moderate to severe chronic periodontal disease, had at least 20 natural teeth, with >10 sites with PD >4 mm	Initial: 39 patients Final: 38 patients	14M/24F
Wu et al. (2022) (25)	China	RCT (Two groups)	Systemically healthy individuals aged between 20 and 60 years. Gender eligible, based on a male: female ratio of around 1:1. Anterior and posterior teeth in both dental arches. No periodontal treatment in the 12 months prior to recruitment. The presence of moderate to severe chronic periodontitis, with three or more quadrants in the oral cavity presenting at least three sites with residual PD ≥5 mm with or without BOP during re-evaluation after initial periodontal therapy	Initial: 40 patients Completed the study: 37 patients	15M/22F
Graetz et al. (2022) (26)	Germany	RCT (Split mouth, Pilot)	Age ≥18 and ≤70 years, recently diagnosed stage III or IV generalized periodontitis according to the 2018 classification, ≥16 scorable teeth without root caries (diagnosed via radiographs and by clinical visual and tactile methods), availability for NSPT and reevaluation during the next 4 ± 1 months, no physical or mental impairment, not taking medications that influence salivary flow, no special dietary restrictions, and an informed consent to be treated during dental education	Initial: 23 patients Final: 20 patients	10M/10F
Avradopoulos et al. (2004) (16)	USA	RCT (Split mouth match site design, Pilot)	Patients with chronic periodontitis and who completed initial periodontal therapy and periodontal maintenance at least one year prior to initiation into the study	Initial: 7 patients Final: 6 patients	5M/2F
Yuryevna et al. (2023) (27)	Russia	Clinical study (Two groups)	Patients with chronic generalized periodontitis of moderate to severe degree, non-smokers, no generalized pathology, no orthopedic and orthodontic treatment	30 patients	13M/17F

RCT, randomized clinical trial; SRP, scaling and root planing; PD, probing depth; mm, millimeters; BOP, bleeding on probing; NSPT, non-surgical periodontal therapy; M, male; F, female.

### 3.3 Clinical intervention and outcomes assessed

The intervention investigated in these clinical trials was the adjunctive use of periodontal endoscopy in non-surgical periodontal therapy, SRP, compared to SRP alone as the control. Different clinical outcomes were assessed across the included studies, all of which were considered as outcome variables in this systematic review. Those outcomes involved probing depth, clinical attachment loss, bleeding on probing, gingival index, treatment time, and percentage of residual calculus, along with other clinical parameters. Comprehensive information on the intervention, control, and outcomes measured is illustrated in Table 4.

**Table 4:** Comprehensive information on the intervention, control, and outcomes.

Author, Year	Treated sites	Teeth type	Study parameters/outcomes	Control details	Intervention (test) details	Follow up time	Outcomes	Main conclusion
Wright et al. (2023) (23)	Initial: 3,864 3 months: 3,573 6 months: 2,912 12 months: 1,794	Both single-rooted and multi-rooted teeth	PD, CAL, FGM, BOP	SRP using conventional loupe magnification with 2.5 magnification and a headlamp using an ultrasonic scaler and Gracey curettes under local anesthesia, calculus removal and smooth surfaces were checked using a 11/12 explorer	SRP using a periodontal endoscope, completed in two sessions by a single experienced dental hygienist, using only ultrasonic instruments for supra- and sub-gingival debridement with enhanced visualization via a periodontal endoscope under local anesthesia, calculus removal and smooth surfaces were checked using a 11/12 explorer	1, 3, 6, 12 months	Overall, when clinical parameters from all teeth were considered (PD, CAL, FGM, BOP), the percentage of sites displayed improvement was similar between control and intervention groups. However, at multirooted sites, maxillary multirooted interproximal sites favored the periodontal endoscope at the 3- and 6-month periods (*P* = 0.017 and 0.019, respectively) in terms of the percentage of sites with improved CAL. Mandibular multirooted interproximal sites showed more sites with improved CAL using conventional SRP than with the periodontal endoscope (*P* < 0.05). PD, BOP, and FGM were comparable.	The use of a periodontal endoscope was more beneficial in multirooted sites compared to single-rooted sites, specifically in maxillary multirooted sites
Michaud et al. (2007) (17)	35 tooth pairs (70 teeth total)	Multi-rooted first or second molars with non-fused roots	Percentage of residual calculus, treatment time	SRP with hand instruments and ultrasonic tips until a smooth root surface was attained and no residual calculus detected with a number 3 cow horn explorer, maximum treatment time was set at 60 min	SRP with ultrasonic tips and hand instruments with adjunctive use of PE, the endpoint was no visible calculus (visualized on the LCD screen) remaining on the root surface, maximum treatment time was set at 60 min per tooth	Immediate extraction	The total percentage of residual calculus did not differ significantly between test and control groups. The percentage of residual calculus for Interproximal PD ≤6 mm was statistically significant difference (12.70%–5.28% for nPE and 10.25%–4.48% for PE) and not statistically significant difference for interproximal sites with PD >6 mm. The percentage of residual calculus for facial/lingual surface sites ≤5 mm or >5 mm was statistically insignificant. The percentage of residual calculus for shallow vs. deep furcation involvement was statistically insignificant. The use of PE resulted in longer treatment time. Mean treatment time per tooth was 19.34 ± 8.75 min. for PE and 13.6 ± 5.3 for control. The difference of 5.74 ± 6.86 was statistically significant	The use of the PE as an adjunct to traditional SRP provided no significant improvement in calculus removal in multi-rooted molar teeth and resulted in longer treatment time
Blue et al. (2013) (14)	202 test sites, 162 control sites	NA	PD, CAL, BOP, GI and calculus index	SRP without the use of PE, an ODU 11/12 explorer used for tactile detection of calculus and ascertaining completion of root planing, the time allotted depended on the amount of sub-calculus and its subsequent removal by the clinician	SRP with the use of PE, an ODU 11/12 explorer used for tactile detection of calculus and ascertaining completion of root planning with the aid of PE, the time allotted depended on the amount of sub-calculus and its subsequent removal by the clinician	6–8weeks, 3 months	PD reduction and CAL gain difference between PE and nPE were statistically insignificant. Mean changes BOP was greater and statistically significant for PE sites when compared to nPE sites. Mean change in the GI was greater and statistically significant for PE sites. A statistically significant difference in calculus detection between nPE and PE quadrants, favoring PE.	The adjunctive use of the PE improved periodontal outcomes with respect to gingival inflammation and BOP, however, it was not found to be superior to traditional SRP with regard to PD reduction and CAL
Geisinger et al. (2007) (15)	50 tooth pairs (100 teeth total)	Single-rooted teeth	Percentage of Residual Calculus, treatment time	SRP by single clinician with sharp hand instruments and ultrasonic scalers until a smooth, hard root surface was attained with no calculus detected with a number 3 cow horn explorer	SRP by single clinician with sharp hand instruments and ultrasonic scaler with PE simultaneously to visualize residual calculus on the root surface with endpoint of therapy was no visible calculus, maximum treatment time was set at 40 min per tooth	Immediate extraction	The total percentage of residual calculus for all surfaces was statistically significant (17.84%–3.81% for nPE teeth and 15.70%–2.75% for PE teeth). The use of PE resulted in a statistically significant longer treatment time, average treatment time per tooth was 19.88 ± 6.37 min. for PE while 13.76 ± 4.08 min. for control.	The use of the PE resulted in a statistically significant overall improvement in calculus removal during SRP in deep PD >4 mm, however, no statistically significant difference in shallow PD
Naicker et al. (2022) (24)	NA	Both single and multirooted teeth	PD, CAL, BOP, gingival recession, PI, RBL	SRP with hand and powered instruments, 1.5 h for each quadrant under local anesthetic	SRP with hand and powered instruments plus PE which was inserted into pockets to detect deposits during initial debridement, 1.5 h for each quadrant under local anesthetic	3, 12 months	At 12 months, the mean PD significantly lower in the PE group (2.70 ± 0.2 mm) compared to control (2.98 ± 0.4 mm) while no difference at 3 months. BOP% was significantly lower in the PE group at baseline, 6, 9, and 12 months. PI percentage was reported to be significantly lower in the PE group at baseline, 9 and 12 months. The mean change in RBL was higher in the PE group with more gain (0.69 ± 0.3 mm) as compared with the control group (0.49 ± 0.2 mm) (*p* < 0.05). CAL did not differ between PE and nPE (*p* = 0.3), similarly mean recession (*p* = 0.5)	The adjunctive use of the PE provided a slight statistically significant benefit to the outcomes of non-surgical therapy particularly at deeper PD
Wu et al. (2022) (25)	830 test sites, 799 control sites	NA	PD, BOP, CAL, PI	SRP was carried out using an EMS Piezon® Mini-Master ultrasonic scaler and hand curettes by the same periodontist, treatment terminated when the operator was satisfied that the root surfaces were smooth and thoroughly debrided as assessed using a dental explorer (EXD 11–12, Hu-Friedy®)	SRP was carried out using an EMS Piezon® Mini-Master ultrasonic scaler and hand curettes with the aid of PE by the same periodontist, treatment terminated when no calculus could be seen under PE	3, 6 months	Sites treated with SRP + PE presented a statistically significant increased reduction in PD at both 3 and 6 month with statistically significant lower PI means compared to control sites. No significant differences in CAL or BOP between the two groups at either follow-up	SRP + PE resulted in significant reductions in PD and PI compared to SRP alone in residual pockets with a PD ≥5 mm. PE is a promising therapeutic concept in the treatment of persisting pockets
Graetz et al. (2022) (26)	1,362 test sites, 1,380 control sites	Both single-rooted and multi-rooted teeth	BOP, PD, CAL, presence of hard calculus deposits, treatment time	SRP with air pressure sonic scaler and hand instruments (Gracey curettes) under local anesthesia by two investigators, the clinical endpoint of treatment was defined as the time when clean (smooth) root surface was perceived using the periodontal probe, tips of the sonic scaler, and/or curettes	SRP with air pressure sonic scaler and hand instruments (Gracey curettes) with PE under local anesthesia by two investigators, the clinical endpoint of treatment was defined as the time when no hard calculus deposits were detectable visually in PE image	4 ± 1 months	For CAL and PD, a statistically significant greater attachment gain and a higher PD reduction were observed in the nPE group. PD of 4–6 mm showed significantly higher CAL gain in the nPE group. PD reduction and CAL gain in the lower jaw were significantly higher in the nPE group. No significant difference in BOP at patient's level between groups, however, at tooth level, significantly fewer teeth surfaces with a positive BOP in the nPE group. Significantly longer time for PE and more surfaces with hard calculus deposits in PE at baseline	No clinical benefits were observed at the patient's level for the additional use of periodontal endoscopy in NSPT within four months of observation
Avradopoulos et al. (2004) (16)	22 test sites, 22 control sites	Both single-rooted and multi-rooted teeth	PI, GCF, GI, PD, CAL, BOP	SRP using ultrasonic scalers by single clinician (dental hygienist), treatment timed and set approximately eight minutes per site	SRP using ultrasonic scalers and PE by single clinician (dental hygienist), time allotted depended on amount of subgingival calculus seen in PE and its removal by the clinician	1, 3 months	Changes were not significant between nPE and PE in PD reductions, PI GI, BOP, CAL, and GCF inflammatory markers (IL-1B, PGE2)	The implementation of PE did not show statistically significant results when compared to traditional SRP
Yuryevna et al. (2023) (27)	NA	NA	Green-Vermillion hygienic index (OHI-S), papillary-marginal-alveolar index (PMA) BOP, PD	SRP, the classical method	SRP using PE	1 month	PD in the PE group before treatment was 6.2 ± 0.2 mm, after treatment – 3.5 ± 0.4 mm while in nPE group PD was 5.9 ± 0.4 mm before treatment, after treatment it changed to 4.2 ± 0.2. In PE bleeding decreased by 48%, and in the nPE group only by 22%. PMA index change in PE group 42.5 ± 3.5%–30 ± 2% while in nPE 44.5 ± 2.5%–6.0 ± 1.0%. OHI-S improved in both groups, in PE 3.45 ± 0.2–1.2 ± 0.1 while in nPE 3.41 ± 0.23–1.75 ± 0.1	The use of PE with calculus removal and root polishing undoubtedly leads to a reduction in clinical symptoms and residual calculus compared to conventional treatment

PD, probing depth; CAL, clinical attachment level; FGM, free gingival margin; BOP, bleeding on probing; SRP, scaling and root planing; PE, periodontal endoscopy; *n*PE, no periodontal endoscopy; mm, millimeter; NA, not available; GI, gingival index; min, minutes; PI, plaque index; RBL, radiographic bone level; NSPT, non-surgical periodontal therapy; GCF, gingival crevicular fluid.

Table 5 represents the reported probing depth and residual calculus in all the studies, as both are considered the main parameters in assessing the efficiency of SRP. Probing depth is considered the commonly used clinical parameter indicating the resolution of deep periodontal pockets and restoration of periodontal health, while the amount of residual calculus is a critical indicator for accurate debridement necessary for arresting inflammation and disease progression. The corresponding *p*-values were also illustrated in the table, providing a clear appraisal of the significance of the observed difference between the two investigated groups.

**Table 5:** Main clinical outcomes (probing depth and residual calculus) among the included studies.

Author (year)	Clinical parameter	SRP + PE (test)	SRP alone (control)	*P*-value (sig.)
Wright et al. (2023) (23)	Residual calculus (%)	12.32 ± 4.09	13.47 ± 4.92	0.097 (N)
Michaud et al. (2007) (17)	Residual calculus (%)	15.70 ± 2.75	17.84 ± 3.81	<0.001 (Y)
Blue et al. (2013) (14)	Probing depth (mm)	3.55 ± 0.8	3.83 ± 1.2	0.1710 (N)
Geisinger et al. (2007) (15)	Probing depth (mm)	2.7 ± 0.2	2.98 ± 0.4	0.02 (Y)
Naicker et al. (2022) (24)	Probing depth (mm)	3.12 ± 0.63	4.0 ± 0.68	0.001 (Y)
Wu et al. (2022) (25)	Probing depth (mm)	NR	NR	0.038 (Y, favoring nPE)
Graetz et al. (2022) (26)	Probing depth (mm)	4.0 ± 1.54	3.68 ± 1.43	0.76 (N)
Avradopoulos et al. (2004) (16)	Probing depth (mm)	3.5 ± 0.4	4.2 ± 0.2	NR
Yuryevna et al. (2023) (27)	Probing depth (mm)	NR	NR	0.871 (N)

SRP, scaling and root planing; PE, periodontal endoscopy; %, percentage; mm, millimeters; NR, not reported; Sig, significance; N, no; Y, yes; *n*PE, no periodontal endoscopy.

### 3.4 The efficacy of the adjunctive use of periodontal endoscopy

The results across the reviewed RCTs included in this study were diverse, with no clear consensus on the adjunctive use of periodontal endoscopy with SRP vs. conventional SRP alone. Some studies reported that this adjunctive use of periodontal endoscopy resulted in statistically significant better clinical outcomes, mainly probing depth and clinical attachment level (15, 24, 25, 27), particularly in sites with deep periodontal probing depth (15, 24) and maxillary multi-rooted teeth (23). Conversely, other studies reported no additional benefit from the adjunctive use of periodontal endoscopy, as no significant clinical difference was detected between the investigated methods, particularly in shallow sites where outcomes were comparable (14–17). Notably, one study reported superior clinical outcomes, greater attachment gain, more probing depth reduction, and less bleeding on probing in the conventional non-surgical periodontal therapy group with SRP alone compared to the adjunctive use of periodontal endoscopy, suggesting that periodontal endoscopy may not universally enhance treatment efficacy (26). Although the adjunctive use of periodontal endoscopy appears to contribute favorably to calculus detection with less residual calculus after debridement in one study (15), no significant difference was reported in another study (17). However, its implementation seems more time-consuming and utilizes greater treatment time (15, 17).

### 3.5 Quality assessment and risk of bias

**Figure 2:**
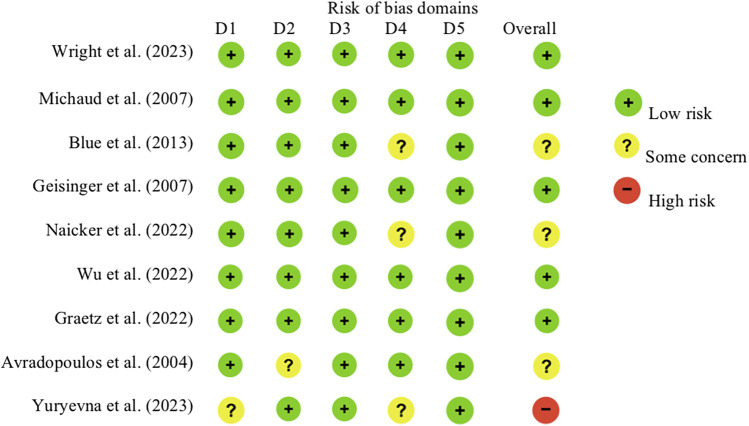
Risk of bias assessment utilizing the RoB2: the revised Cochrane risk of bias tool for RCTs.

Following the RoB2, the revised Cochrane risk of bias tool for RCTs, five studies were assessed to possess a low risk of bias (15, 17, 23, 25, 26), three studies had some concern (14, 16, 24), while one study was deemed to feature a high risk (27). Figure 2 provides detailed information on the five domains of each study.

### 3.6 Results of meta-analyses

Meta-analysis was implemented to assess the efficacy of non-surgical therapy with or without the adjunctive use of periodontal endoscopy, in particular the changes in probing depth, the amount of residual calculus, and treatment time with a random effect model.

**Figure 3:**
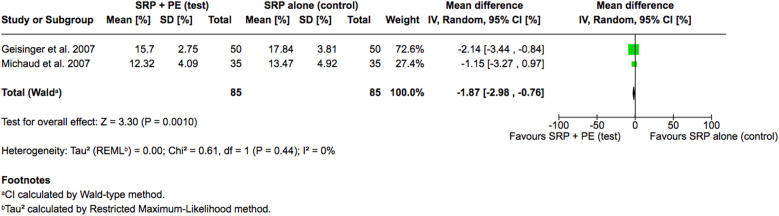
Forest plot of the percentage of residual calculus, comparing SRP with the aid of periodontal endoscopy vs. SRP alone.

When investigating the amount of residual calculus, only two of the included studies reported the amount of residual calculus as an outcome (15, 17). The meta-analysis revealed that the adjunctive use of periodontal endoscopy significantly outperformed the conventional non-surgical periodontal therapy with SRP alone, with a pooled mean difference of −1.87% (95% CI: −2.98% to −0.76%, *p* = 0.0010), indicating a clear benefit. Notably, this result was highly consistent with no observed heterogeneity between the studies (I^2^ = 0%, *p* = 0.44) (Figure 3). Overall, the results provide reliable evidence supporting the efficacy of periodontal endoscopy as an adjunct to conventional non-surgical periodontal therapy in reducing the amount of residual calculus.

**Figure 4:**
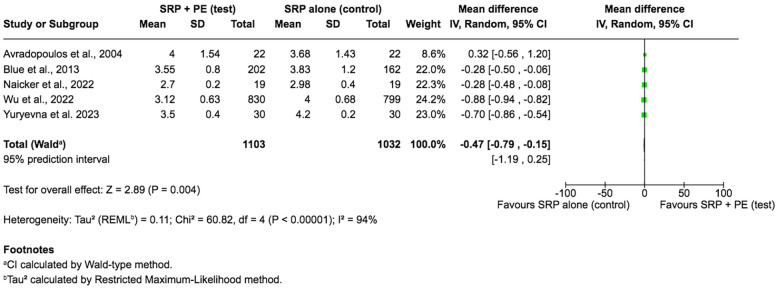
Forest plot of the changes in probing depth, comparing SRP with the aid of periodontal endoscopy vs. SRP alone.

Regarding the changes in probing depth, four studies were omitted from the meta-analysis owing to the reasons listed: probing depth was not an outcome in two studies (15, 17), and the other two studies failed to provide the exact mean change in the probing depth; only *p*-values were reported (23, 26). Thus, only five studies were deemed eligible for the meta-analysis. The results disclosed a statistically significant advantage of the adjunctive use of periodontal endoscopy over the conventional non-surgical periodontal therapy with SRP alone, with a pooled mean difference of −0.47 (95% CI: −0.79 to −0.15, *p* = 0.004). However, the results displayed a high heterogeneity (I^2^ = 94%, *p* < 0.00001) (Figure 4), indicating considerable variability in results. Moreover, the wide prediction interval implies that the true effect size may vary, and future studies might not always replicate this effect. Therefore, while the adjunctive use of periodontal endoscopy appears beneficial overall in probing depth change, the variability across studies warrants cautious interpretation.

**Figure 5:**
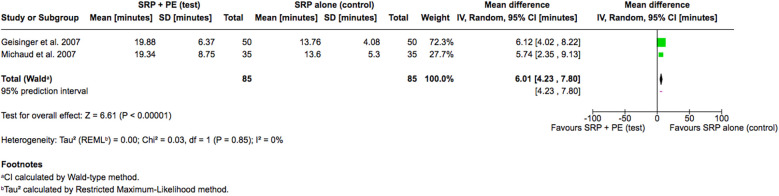
Forest plot of the utilized treatment time, comparing SRP with the aid of periodontal endoscopy vs. SRP alone.

Lastly, considering treatment time, only two studies reported mean treatment time as an outcome and thus underwent meta-analysis (15, 17). The result revealed that the adjunctive use of periodontal endoscopy significantly increased treatment time by a mean difference of 6.01 min (95% CI: 4.23–7.80, *P* < 0.00001) (Figure 5) compared to SRP alone. Both studies exhibited similar direction and magnitude of effect, with no statistical heterogeneity observed (I^2^ = 0%). This suggests reliable evidence the adjunctive use of periodontal endoscopy will usually require longer treatment time.

## 4 Discussion

Bacterial plaque and calculus deposits are the primary microbiological etiological factors implicated in periodontal inflammation and tissue breakdown. Accordingly, periodontal therapy seeks to eradicate these contributing factors to establish a biologically compatible root surface. However, it is significantly difficult to completely remove subgingival bacterial plaque and especially calculus deposits with conventional non-surgical periodontal therapy, SRP. This becomes more challenging as the periodontal probing depth increases (28). While open flap debridement enhances visibility and provides better access to root surfaces, leading to superior efficacy in calculus removal, this surgical approach presents some drawbacks compared to conventional non-surgical therapy; these include postoperative pain, discomfort, surgical complications, longer healing periods, and possible soft tissue recession with subsequent root surface exposure (29). This might discourage patients from opting for this surgical option, favoring the conventional non-surgical SRP.

With the advancement of technology, adjunctive treatment modalities have emerged in periodontology; one of these is the periodontal endoscopy, which was introduced by Stambaugh et al. (30). They inferred endoscopic images of the periodontal structures and stated that the periodontal endoscope allowed for immediate, live display with magnified images of the subgingival area, root surface, soft tissue, and calculus deposits, which could assist clinicians with adequate treatment. Several studies have investigated the implementation of periodontal endoscopy in comparison to the conventional approaches in subgingival assessment and debridement (12, 14–17, 23–27). Periodontal endoscopy outperformed the conventional dental explorer in residual calculus detection, particularly in multirooted teeth and proximal surfaces (12). Ultimately, leading to more effective debridement and better treatment outcomes (15, 24, 25, 27).

Although few RCTs have investigated the adjunctive use of periodontal endoscopy in non-surgical periodontal therapy as opposed to the conventional approach, SRP alone, the findings have been inconclusive with no consensus on its impact on different clinical outcomes. This may have hindered the widespread adoption and application of periodontal endoscopy into clinical settings. Hence, this systematic review and meta-analysis was executed to critically appraise the available evidence and determine whether the adjunctive use of periodontal endoscopy offers a superior clinical outcome over the conventional SRP alone.

The findings of the systematic review revealed discrepancies among the investigated clinical parameters. Geisinger et al. declared that periodontal endoscopy enhanced calculus removal with significantly less residual calculus after debridement (15). In contrast, Michaud et al. found no significant clinical advantage when employing periodontal endoscopy over the conventional SRP alone (17). Despite these contradictory outcomes, both studies consistently noted that periodontal endoscopy was more time-consuming, requiring significantly longer treatment time. When investigating the changes in periodontal probing depth, the reported results were inconsistent across the studies. Naicker et al. and Wu et al. observed a significant reduction in probing depth with the adjunctive use of periodontal endoscopy (24, 25). In contrast, Blue et al., Avradopoulos et al., and Wright et al. discovered no significant difference (14, 16, 23). Remarkably, Graetz et al. reported significantly greater reduction in probing depth with the conventional therapy, SRP alone (26). These conflicting findings highlighted the need for a meta-analysis for a more comprehensive and objective assessment of the evidence.

This meta-analysis verified a statistically significant superior outcome of the adjunctive use of periodontal endoscopy in calculus removal. This improvement could be attributed to the enhanced visualization of the root surface due to the direct magnified imaging displayed on the endoscopy screen during treatment, thereby facilitating more efficient debridement vs. conventional SRP alone, with strong and highly reliable evidence reinforcing the clinical value of the aid of periodontal endoscopy in SRP. Clear and reliable evidence was also revealed regarding treatment time; the meta-analysis reinforced that the adjunctive use of periodontal endoscopy in non-surgical periodontal therapy will require significantly longer treatment time. This can be linked to the superior detection of subgingival calculus with the aid of the periodontal endoscopy, necessitating more time for thorough removal. However, it is important to recognize that periodontal endoscopy seems to present a learning curve. Studies have exhibited that as clinicians gain experience and proficiency with the device, the treatment duration progressively decreases, approaching those utilized with the conventional SRP alone (15, 17). Therefore, while initially more time-consuming, this time factor should not be reflected as a constraint to the application of periodontal endoscopy. However, a possible constraint of its clinical application is the associated costs, not only the expense of the equipment itself but also the ongoing maintenance fees and the time and financial investment required for training. Probing depth change was statistically significant, favoring the adjunctive use of periodontal endoscopy, as shown in the meta-analysis. However, a very high level of heterogeneity was observed, suggesting substantial variability in probing depth changes outcome across the studies. This was the case in the included studies, as high variability in probing depth outcome was noted. This could be attributed to several factors, including population differences, operator experience and proficiency, variations in follow-up durations, and discrepancies in sample size among the studies. Given this considerable variation, the interpretation of the probing depth outcome should be approached with caution.

The most recent systematic review on this topic, published in 2023 by Ardila and Vivares-Builes, included only three RCTs with a search period up to January 2023 with no incorporation of a meta-analysis (31). Only one meta-analysis prior to this was published in 2017 by Kuang et al. and had five studies that underwent quantitative assessment (32). In contrast, this systematic review and meta-analysis, which involves nine RCTs, offers a more comprehensive evaluation regarding the efficiency of the adjunctive use of periodontal endoscopy in non-surgical periodontal therapy. The structured and well-established protocol of this study strengthens it and enhances the reliability of the reported findings, with the information presented being more robust and trustworthy. Among the other strengths are the larger number of studies with greater sample size compared to the previously published reports, a more expansive search with no starting date or lower limit, longer follow-up durations, and a quantitative meta-analysis of different clinical outcomes. However, some limitations should be noted. The relatively small number of RCTs could reflect the fact that periodontal endoscopy remains a relatively new technique with limited application in clinical dental practice. Additionally, the high heterogeneity observed in the quantitative assessment of one investigated outcome warrants caution in the interpretation of the results of this specific parameter.

## 5 Conclusion

This systematic review and meta-analysis demonstrated that the adjunctive use of periodontal endoscopy in SRP offers distinct advantages as opposed to conventional SRP alone, particularly in terms of more efficient calculus removal, although it appears to be more time-consuming. Yet, more homogenous RCTs are necessary to attain clear evidence on other clinical outcomes and formulate definitive recommendations for its use. The decision to utilize the benefit of this adjunctive technology in non-surgical periodontal therapy should be based on a reasoned case-by-case assessment by clinicians, weighing its benefit alongside SRP.

## Data Availability

Publicly available datasets were analyzed in this study. This data can be found here:
- Wright HN, Mayer ET, Lallier TE, Maney P. Utilization of a periodontal endoscope in nonsurgical periodontal therapy: A randomized, split-mouth clinical trial. J Periodontol. (2023) 94(8):933–943. doi:10.1002/JPER.22-0081. - Michaud RM, Schoolfield J, Mellonig JT, Mealey BL. The efficacy of subgingival calculus removal with endoscopy-aided scaling and root planing: a study on multirooted teeth. J Periodontol. (2007) 78(12):2238–2245. doi:10.1902/jop.2007.070251. - Geisinger ML, Mealey BL, Schoolfield J, Mellonig JT. The effectiveness of subgingival scaling and root planing: an evaluation of therapy with and without the use of the periodontal endoscope. J Periodontol. (2007) 78(1):22–28. doi:10.1902/jop.2007.060186. - Avradopoulos V, Wilder RS, Chichester S, Offenbacher S. Clinical and inflammatory evaluation of Perioscopy on patients with chronic periodontitis. J Dent Hyg. (2004) 78(1):30–38. - Naicker M, Ngo LH, Rosenberg AJ, Darby IB. The effectiveness of using the perioscope as an adjunct to non-surgical periodontal therapy: Clinical and radiographic results. J Periodontol. (2022) 93(1):20–30. doi:10.1002/JPER.20-0871. - Wu J, Lin L, Xiao J, Zhao J, Wang N, Zhao X, et al. Efficacy of scaling and root planning with periodontal endoscopy for residual pockets in the treatment of chronic periodontitis: a randomized controlled clinical trial. Clin Oral Investig. (2022) 26(1):513–521. doi:10.1007/s00784-021-04029-w. - Graetz C, Sentker J, Cyris M, Schorr S, Springer C, Fawzy El-Sayed KM. Effects of Periodontal Endoscopy-Assisted Nonsurgical Treatment of Periodontitis: Four-Month Results of a Randomized Controlled Split-Mouth Pilot Study. Int J Dent. (2022) 2022:9511492. doi:10.1155/2022/9511492. - Yuryevna OL, Andreevich AN, Yuryevna KA. Effectiveness of using endoscopic technique in patients with periodontitis of different degrees. Prac Orient Sci: UAE – RUSSIA – INDIA. (2023) 130. - Blue CM, Lenton P, Lunos S, Poppe K, Osborn J. A pilot study comparing the outcome of scaling/root planing with and without Perioscope™ technology. J Dent Hyg. (2013) 87(3):152–157.
